# Perception of oral hygiene during orthodontic treatment: an eye-tracking and cross-sectional study

**DOI:** 10.1038/s41598-026-63694-7

**Published:** 2026-07-29

**Authors:** Frederic Fuchs, Luisa Krause, Monika Bjelopavlovic, Irene Schmidtmann, Christina Erbe

**Affiliations:** 1 Department of Dentofacial Orthopedics and Orthodontics, University Medical Center of the Johannes Gutenberg-University, Augustusplatz 2, 55131 Mainz, Germany; 2 Department of Prosthetic Dentistry, University Medical Center of the Johannes Gutenberg University, Mainz, Germany; 3 Institute for Medical Biostatistics, Epidemiology and Informatics (IMBEI), University Medical Centre of the Johannes Gutenberg-University, Mainz, Germany

**Keywords:** Oral hygiene, Eye tracking, Orthodontics, Multibracket-appliance, Adolescent, Attin-Index, Diseases, Health care, Medical research

## Abstract

The objective of this study was to analyze visual perception of patients undergoing orthodontic treatment with multi-bracket appliances (MBA), and examine differences in oral hygiene perception among adolescents, their guardians, and dental students. The lay person group included 56 participants (28 adolescents and guardians), while the expert group consisted of 30 dentistry students. Both groups evaluated randomized intraoral frontal photographs of adolescents with MBA, taken with or without a plaque disclosing agent. Using eye-tracking methods, eye movements were recorded during the evaluation. Significant differences in visual path length and oral hygiene evaluation were found. The expert group evaluated oral hygiene most accurately, with parents being stricter and adolescents more lenient. The expert group’s visual path was significantly shorter than the lay person group’s. Use of a plaque disclosing agent improved the accuracy of oral hygiene evaluation (*p* < 0.001). A correlation was observed between oral hygiene behavior and evaluation. In conclusion, dental students provided the most accurate oral hygiene evaluations. Parents were stricter, and adolescents were more lenient. Among adolescents, longer brushing time was weakly associated with more accurate evaluations, and plaque disclosing agents enhanced evaluation accuracy.

## Introduction

In orthodontic treatment, the use of multi-bracket appliances (MBA) and its variations is still widely common despite the existence of modern invisible appliances^1^. Regardless of the treatment method, the most important aim of orthodontic treatment is to achieve an occlusion and bite relation that distribute the load physiologically on the temporomandibular joint and the surrounding structures. From the patient’s perspective, it is of utmost importance that the result go along with a radiant smile, possibly even lifting self-confidence^2,3^.

During treatment, adequate oral hygiene is necessary. Unfortunately, patients might initially experience certain drawbacks during MBA therapy, for example a feeling of insecurity, the wish not to show their teeth and even embarrassment. This discontent may reflect on oral hygiene behavior, resulting in less frequent tooth brushing, reduced diligence and decreased focus on oral hygiene^4,5^. Because wearing the appliance is associated with visits to the orthodontist, and sometimes even painful treatments, patients are less inclined to spend more time on the necessary care for teeth and appliance^6–8^.

Especially at an age where the permanent teeth are present, it is essential to set a path for a thorough and daily oral hygiene. Many are unaware that dentistry is now able to preserve natural teeth well into old age. A failure to uphold a constant oral hygiene level during MBA treatment might lead to an increased risk of caries and periodontal diseases^9^.

Contemporary digital oculometric measurement techniques, particularly eye-tracking methods, are increasingly applied across medical and psychological research, as well as in marketing, computer science, and robotics^10–20^.

The present study employed eye-tracking measurements to examine the perception of oral hygiene during orthodontic treatment, and whether visual perception of oral hygiene differs between adolescents, their parents and dentistry students.

Hence it was the aim of this study to test the following hypotheses:

1. Null hypothesis: There is no difference between adolescents, their parents and dentistry students in their perception of oral hygiene.
2. Alternative hypothesis: There is a difference between adolescents, their parents and dentistry students in their perception on oral hygiene.

Additionally these secondary objectives were set:

1. How does the use of a plaque disclosing agent influence the perception of oral hygiene?
2. How does tooth brushing technique and duration affect the perception of oral hygiene?

## Material and methods

This study took place at the Department of Dentofacial Orthopedics and Orthodontics, University Medical Center of the Johannes Gutenberg-University Mainz, Germany. The participants were separated into two groups: the lay person group consisted of 56 people, of which 28 were adolescents between 12 and 17 years of age, and 28 their parent or legal guardian. All adolescents were selected from the department’s patient’s files. The second group was composed of 30 dentistry students representing the expert group in plaque recognition. To be included in the study, each participant from both groups had to be of general good health and had to have normal eyesight. MBA of the Forestadent brand (Mini Sprint® brackets, 0.022 slot, Forestadent, Pforzheim, Germany) were in situ in all subjects. This study was conducted in full accordance with the ethical principles outlined in the Declaration of Helsinki (1975), as revised in 2013. Ethical approval was obtained from the Ethics Commission of the State Medical Board of Rhineland-Palatinate (Reference no. 10667). Written and verbal informed consent was obtained from all participants and, where applicable, their legal guardians prior to participation.

Intraoral photographs were taken with a Canon EOS D 600 body with the corresponding Canon 100 macro lens and a Sigma EM-140 DG ring flash (Canon, Tokyo, Japan).

**Fig. 1:**
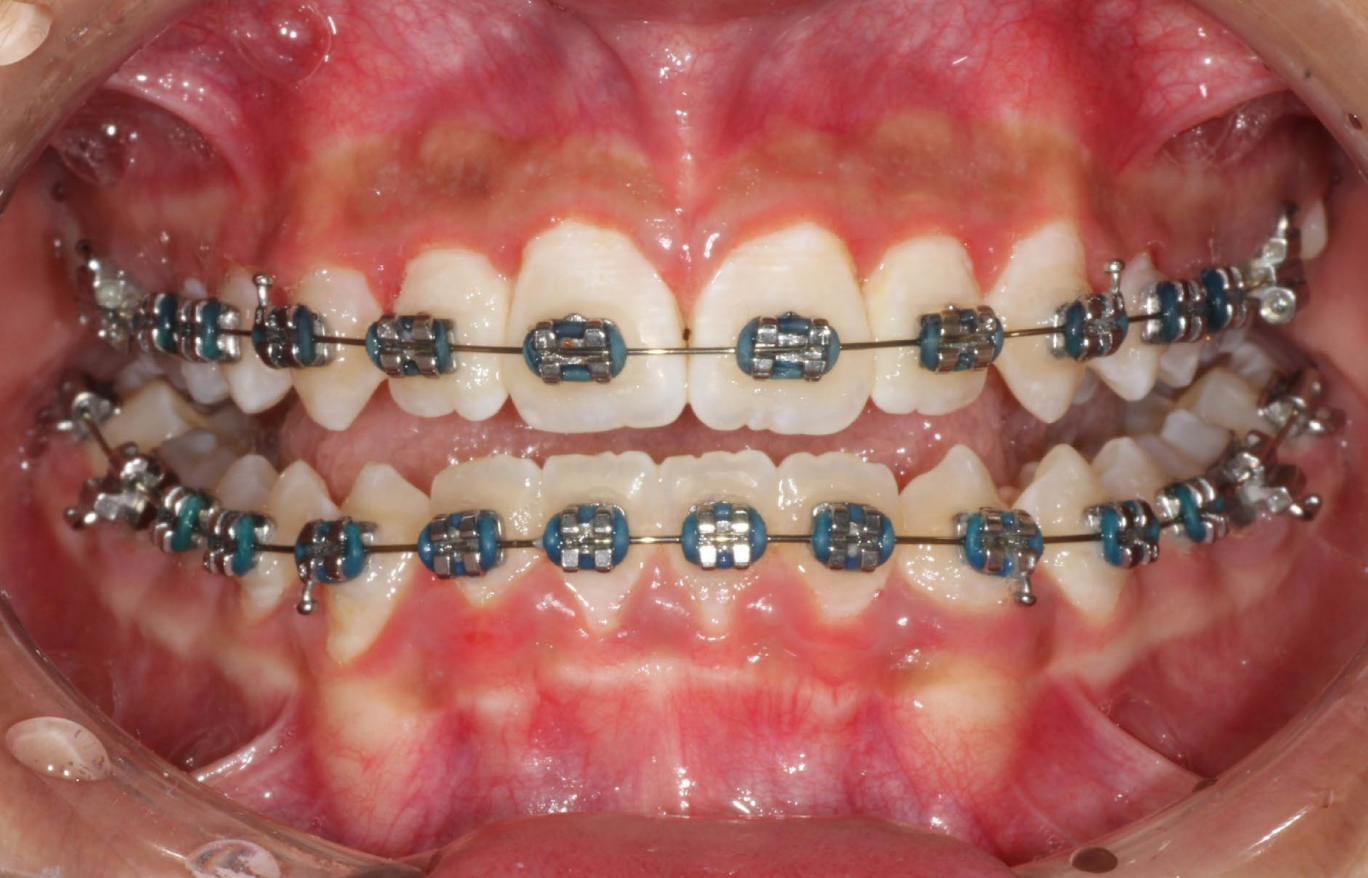
Example photograph of a patient without plaque disclosing agent.

**Fig. 2:**
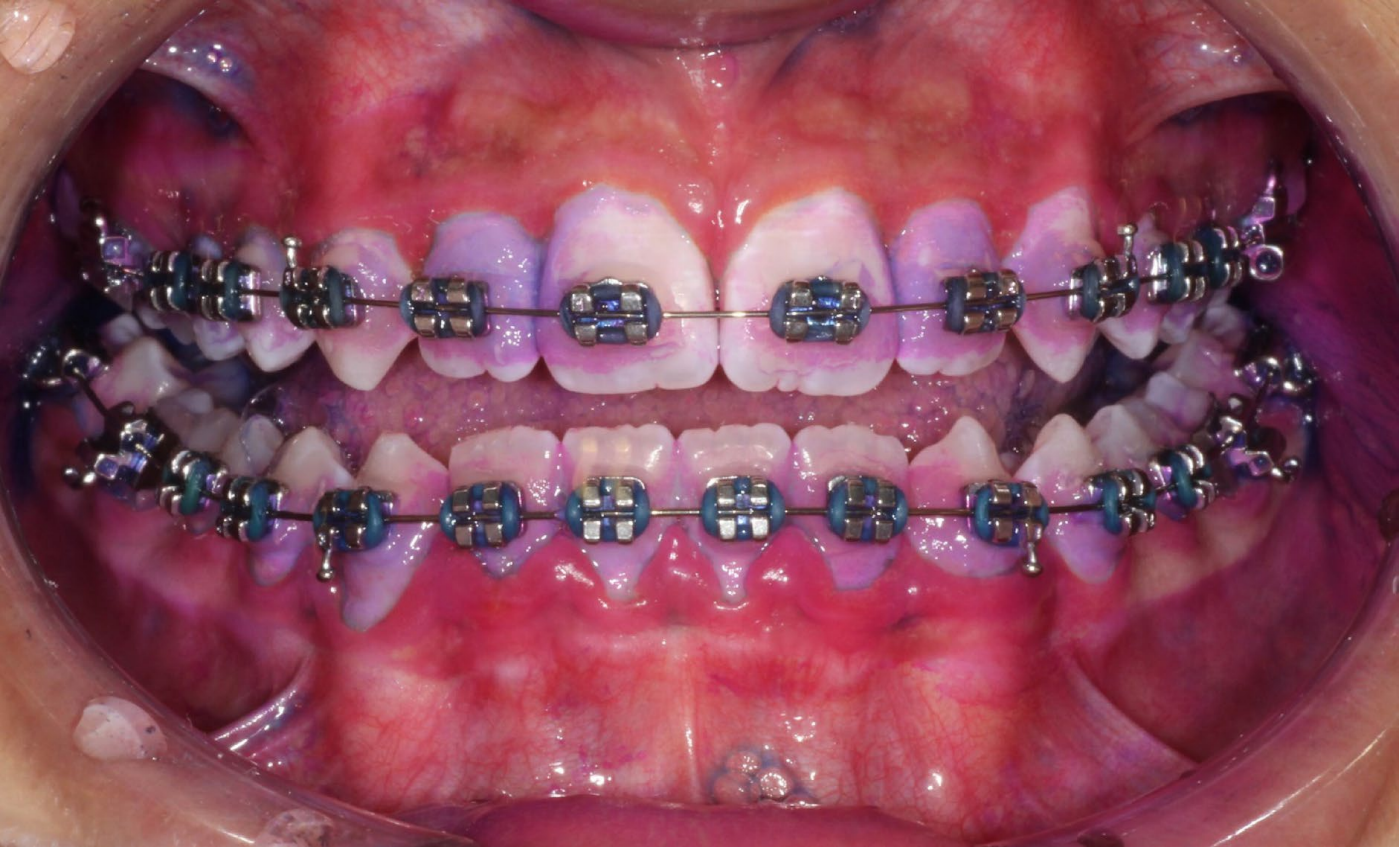
Example photograph of the same patient as in Fig. 1 with plaque disclosing agent.

First, an intraoral frontal photograph was taken without further modification (Fig. 1). After the application of the plaque disclosing agent Mira-2-Ton® (Hager & Werken GmbH, Duisburg, Germany), a second photograph was taken (Fig. 2). Then, the patients were given the chance to brush their teeth at the Department of Dentofacial Orthopedics and Orthodontics’ oral hygiene station, and the brushing technique and duration were documented.

All photographs were cropped uniformly, and variations in brightness, light exposure and orientation were corrected using Photoshop® version 23.11 (Adobe®, San José, California, USA).

After the preparation of the photographs, the areas of interest (AOIs) were determined and an objective evaluation of oral hygiene of the individual adolescent participants was performed by the trained primary investigator.

The objective assessment of oral hygiene was also based on the photos using the Attin index as described by Attin et al.^21^. To make the objective evaluation of the images comparable to the evaluation performed by the test subjects, the plaque index was converted to a scale from 0 to 10 using the following formula:

$${\text{Attin-Index (\% ) = }}\frac{{\frac{Total\;of\;the\;plaque\;values\;of\;each\;anterior\;tooth}{{Measurement\;points}}}}{highest\;grading\;value}{ } \times { 100}$$

“0%” represents no visible plaque, while “100%” represents plaque on every measurement point on every tooth and correspondingly poor oral hygiene.

Afterwards, the recordings were inserted into the eye-tracking system. The eye-tracking system used was the Eyegaze Edge® monocular system (LC Technologies, Fairfax, Virginia, USA), which uses a sampling rate of 60 Hz. It has automatic eye detection and uses the bright-pupil method for detection. According to the manufacturer, the measurement accuracy is < 0.4°, the angular error is specified as 0.3°–0.5°.

**Fig. 3:**
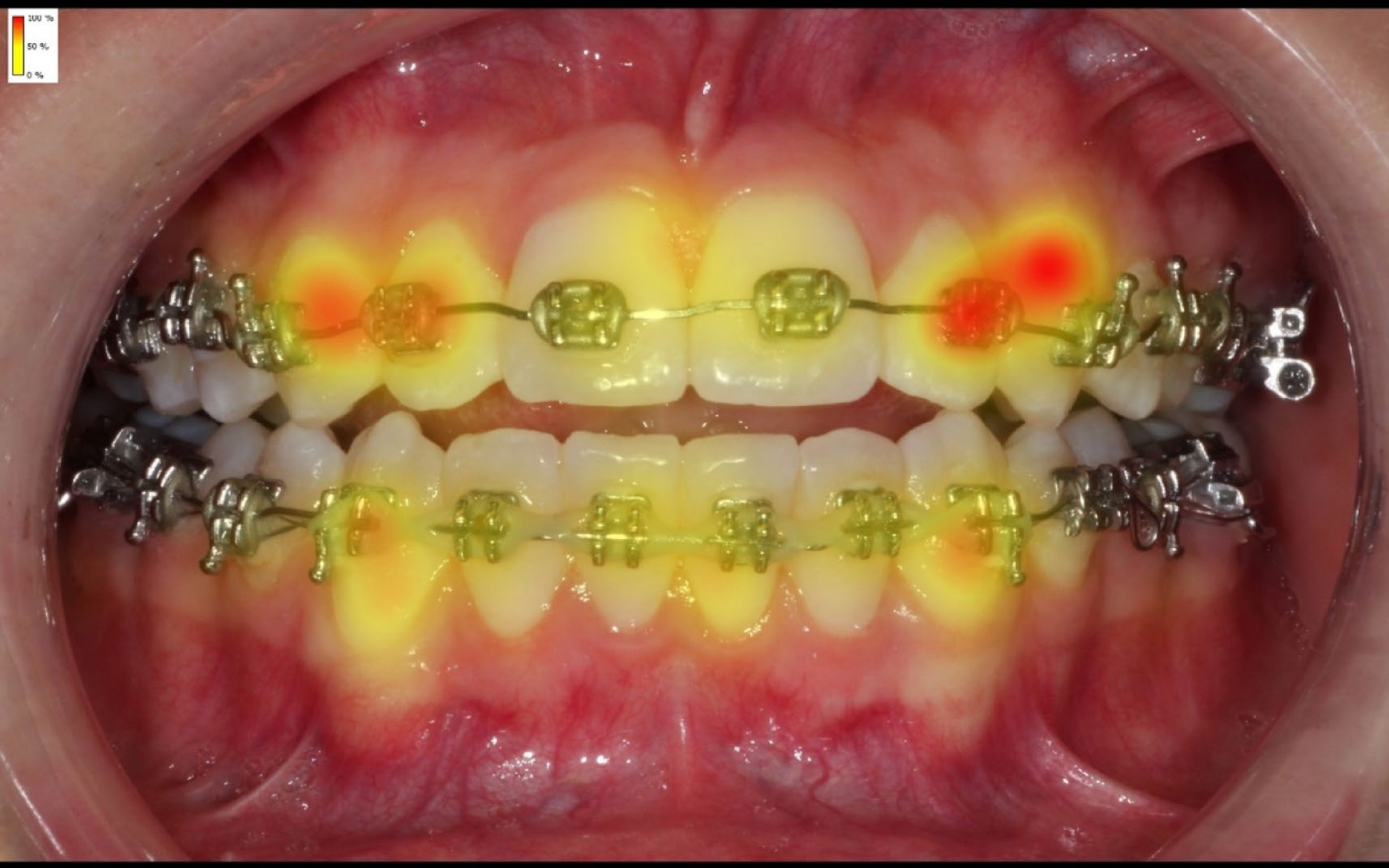
Illustration of the heat map of a test person from the group of students.

**Fig. 4:**
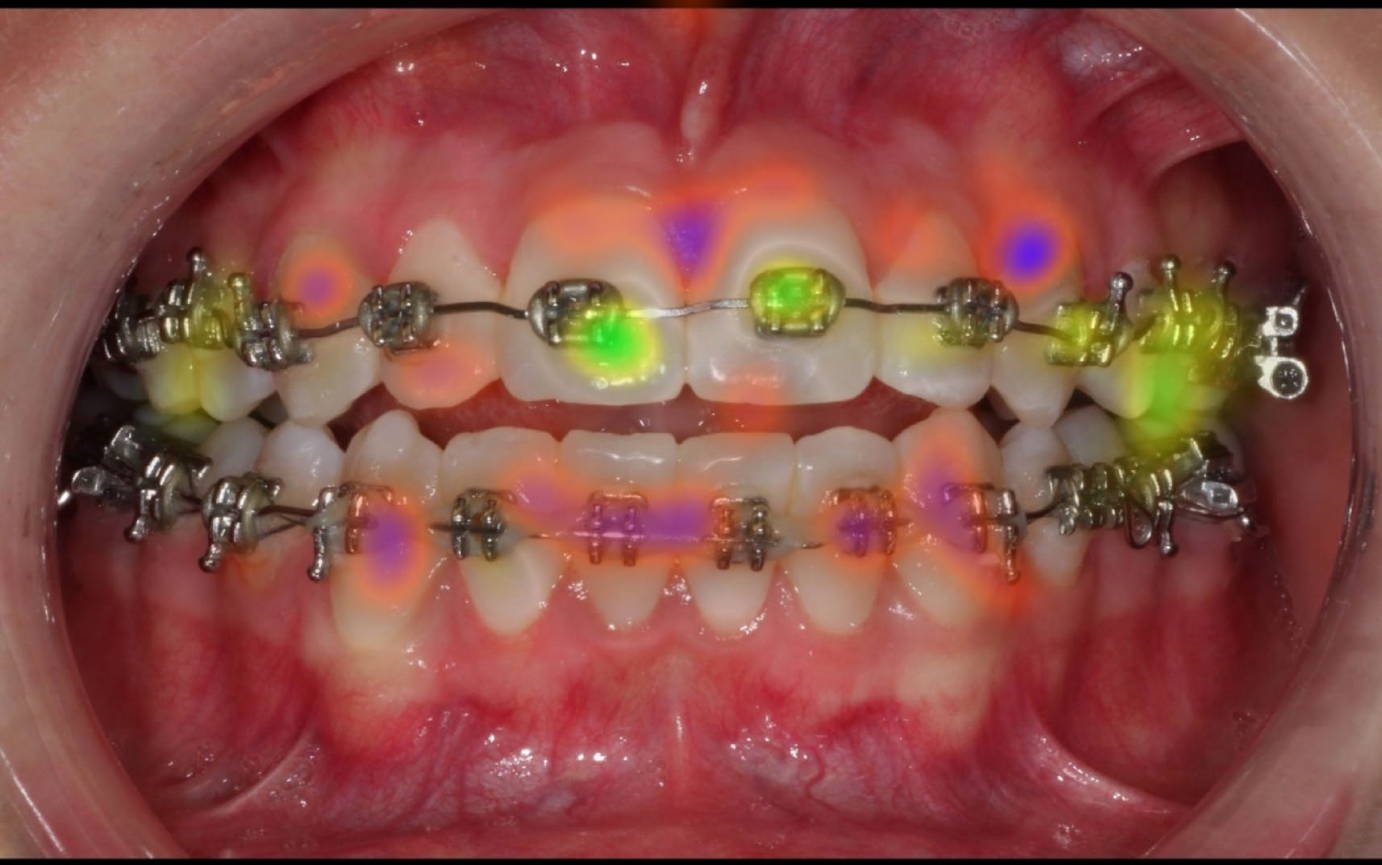
Illustration of the heat map in group comparison, comparison of parents (blue) with young people (yellow).

**Fig. 5:**
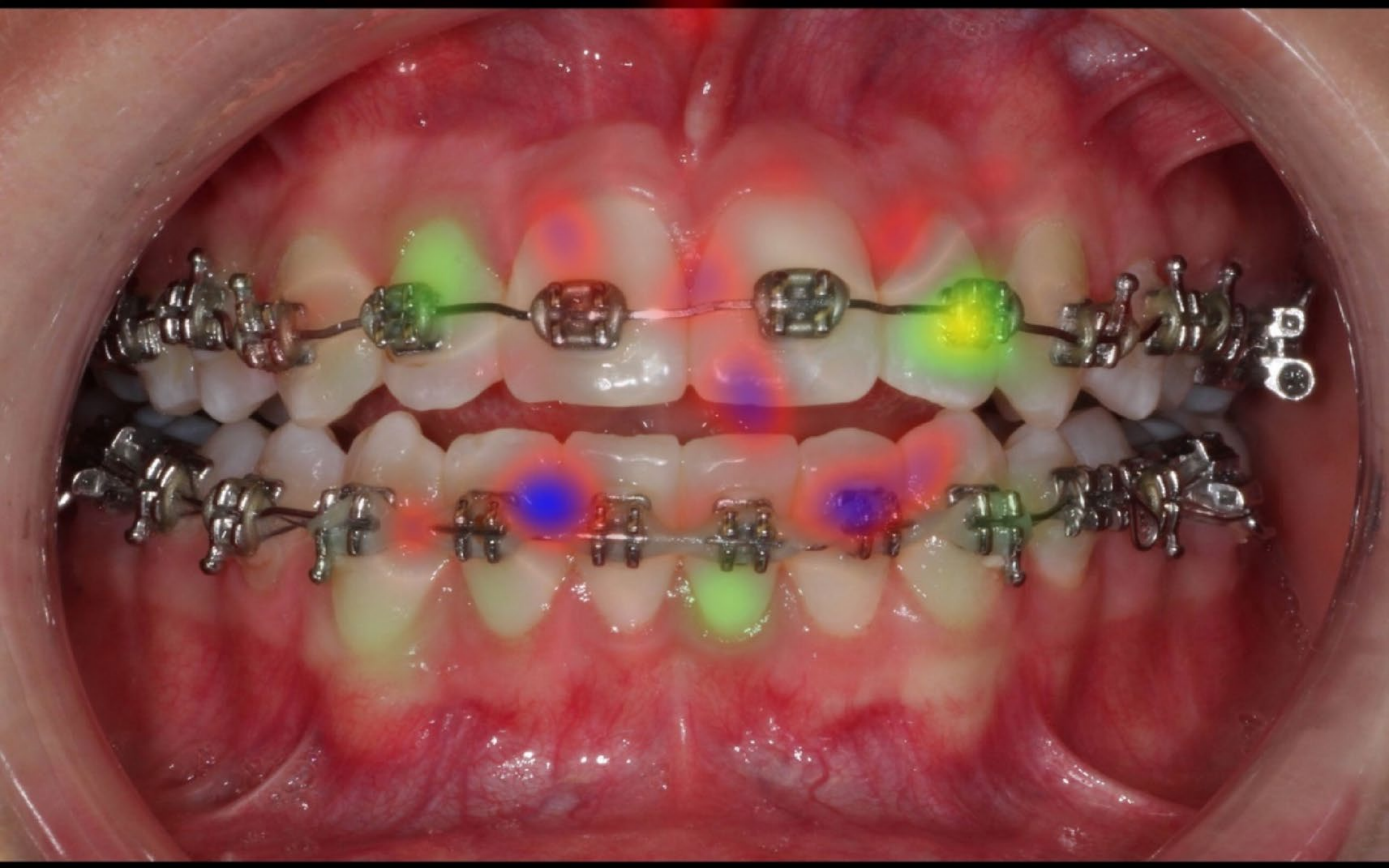
Illustration of the heat map; comparison of parents (blue) with students (yellow).

**Fig. 6:**
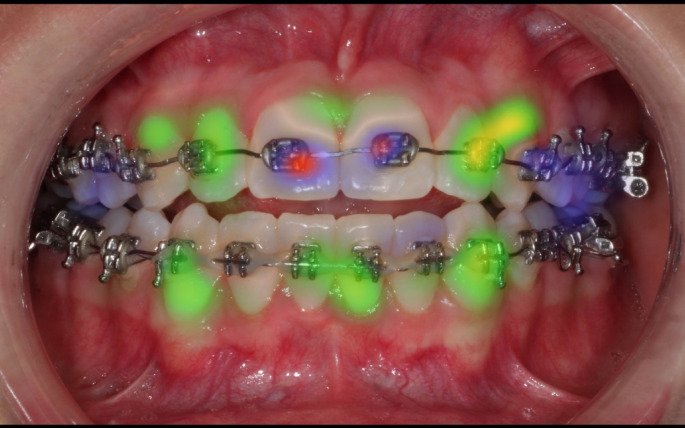
Illustration of the heat map; comparison of young people (blue) with students (yellow).

Both groups were shown all intraoral photographs of the adolescent participants with MBA in a randomized sequence. Participants were shown each image for five seconds, and assessed the status of oral hygiene using a score from 0 (very good) to 10 (very bad). Evaluation scores were communicated acoustically to avoid inaccuracies in the experiment. The eye movements of each person rating the photographs were recorded utilizing the eye-tracking-method. The length of the eye path was recorded from each photo viewed by a test person and the eye tracking data was visualized as a heat map (Figs. 3, 4, 5, 6). However, the visualizations of the raw data were only used for illustration purposes and were not included in statistical analysis. The tables described above with the values for the individual AOIs and the length of the gaze paths were used for this purpose.

### Statistical methods

The primary interest was comparing participant groups, but plaque disclosing agent and an objective measure of oral hygiene were also thought to be relevant for the outcomes. Therefore both, visual path length and evaluation of oral hygiene were analyzed using linear mixed models with participant group (parents, adolescents and students), use of plaque revealing agent and objective evaluation (Attin index) as fixed effects. Participants and photos were considered as random effects. Main effects, two and three factor interactions among the fixed factors were included in the model. Random intercepts were included for both random factors. Further quantitative endpoints were analyzed similarly.

Sample size, i. e. number of participants was chosen for practical reasons. There were pairs of photos (with and without plaque revealing agent) from 30 adolescent patients available. Of these patients 28 with a parent each were willing to participate also in the evaluation of photos. The cohort of students willing to participate amounted to 30. This resulted in 86 participants evaluating 60 photos each.

To the best of our knowledge, formal sample size or power calculation for this design is not available. However, Judd et al.^22^ discuss power considerations for a similar but somewhat simpler design with just one fixed effect at two levels and two random effects. They provide a tool^23^ for power and sample size calculation for this simpler situation. We used this tool to assess whether our sample size would be sufficient to detect meaningful differences.

For this, we considered simplified comparisons, namely comparing two groups of participants evaluating a single type of photos (either with or without plaque revealing agent) ignoring the Attin index. We assumed that the residual variance amounted to 50% of the total variance and that the proportion of the variance due to participants was considerably higher than the proportion of variance due to photos, as the latter was at least partially explained by the objective oral hygiene measure. Therefore, we assumed that variance among participants amounted to 40% of the total variance and the variance among photos amounted to 10%. We only considered random intercepts. Including a total of 56 participants (e. g. comparing 28 parents to 28 adolescents) and 30 photos, a medium effect of d = 0.5 can be demonstrated with 81% power.

For the time to first fixation in an area of interest (AOI) we fitted a proportional hazard model with participant group (parents, adolescents and students), use of plaque revealing agent and objective evaluation (Attin index) as fixed effects and participants and photos as random effects.

For the intra-rater reliability, the Intraclass Correlation Coefficient (ICC) was calculated (0.99). Hence, an inference could be drawn about the reproducibility within the evaluations of the first five patients.

## Results

All participants completed the study.

### Visual path length

The perception of oral hygiene differed between the groups of participants. This was evident especially in the evaluation of oral hygiene by the participants, as well as their visual path length.

The students had the shortest visual path lengths, followed by the adolescents and their parents, who had the longest.

When comparing the length of the visual path and the degree of oral hygiene presented, the length of the visual path decreased from rating level 7 in the native photographs. In the case of photographs with the use of a plaque disclosing agent, there appeared to be shorter gaze path lengths, particularly in the very good evaluation levels 0/1 and in evaluation 9, especially in the students.

**Fig. 7:**
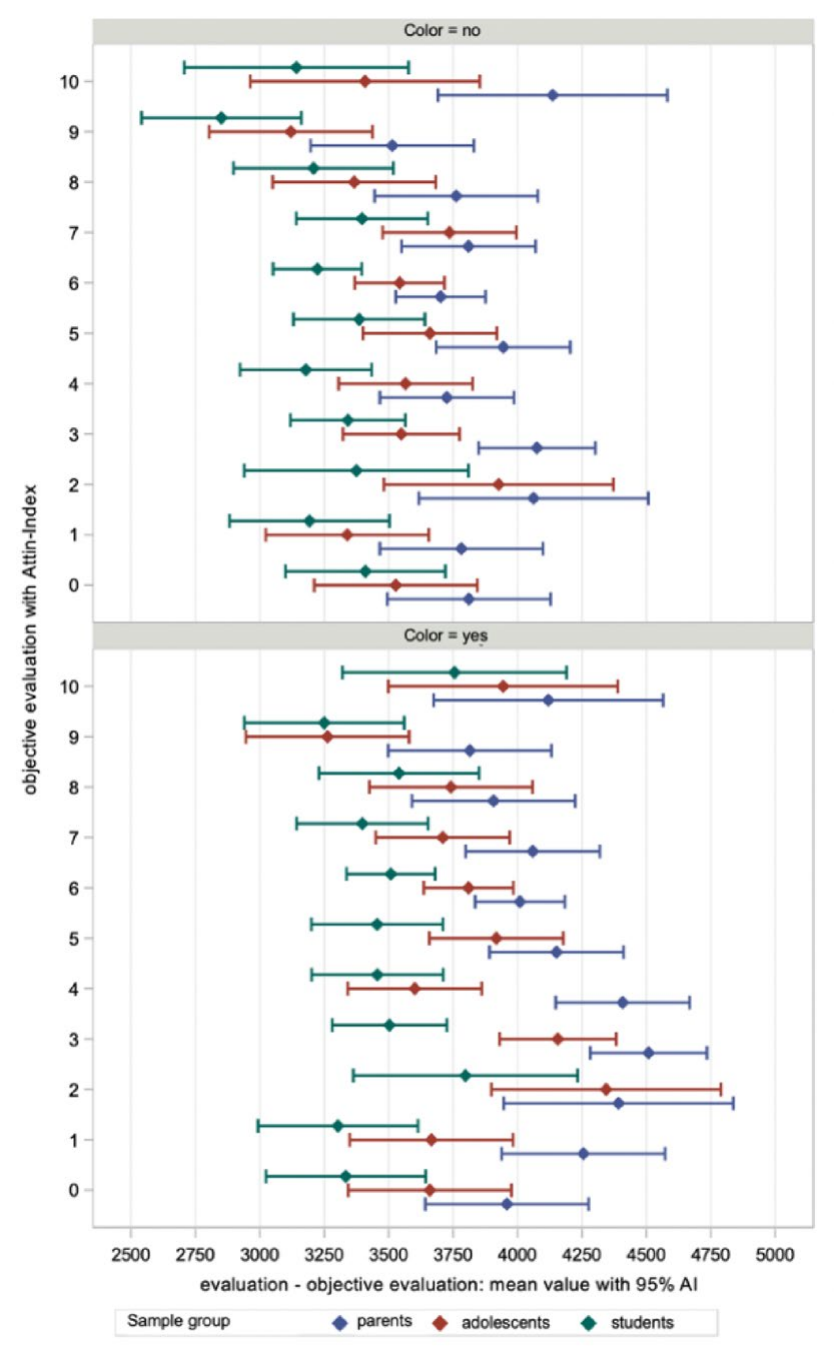
Mean visual path length in pixel with 95%—confidence intervals. Comparison of parents (blue), adolescents (red) and students (green). It was differentiated between records with the use of a plaque disclosing agent (lower) and native records (upper) and sorted according to the objective evaluation of oral hygiene with the Attin-index.

Figure 7 shows that the oral hygiene status, as assessed in the objective evaluation, has a significant influence on the eye path length (*p* = 0.0006, average length between 2943 pxl (95% CI [2693; 3194]) for Attin index = 9 and 3619 pxl (95% CI [3306; 3932.22] for Attin index = 2. The degree of oral hygiene presented therefore was associated with the length of the eye patterns. The use of a plaque disclosing agent was associated with a path longer by 259 pxl (95% CI [214; 305], *p* < 0.0001). The subject group (*p* = 0.0060) also had a significant influence on the eye path length: while mean students’ paths were 2955 pxl (95% CI [2661; 3248]), adolescents had average lengths of 3337 (95% CI [3034; 3640]) and parents 3641 (95% CI [3338; 3944]) There were also significant interactions between Attin index and participant group (*p* = 0.0005) and between Attin and use of plaque disclosing agent (*p* = 0.0026) and even significant three factor interactions (*p* = 0.0014). The differences in visual path length between participant groups were more pronounced when Attin index was higher and with plaque disclosing agent than without (Fig. 7).

### Evaluation of oral hygiene

**Fig. 8:**
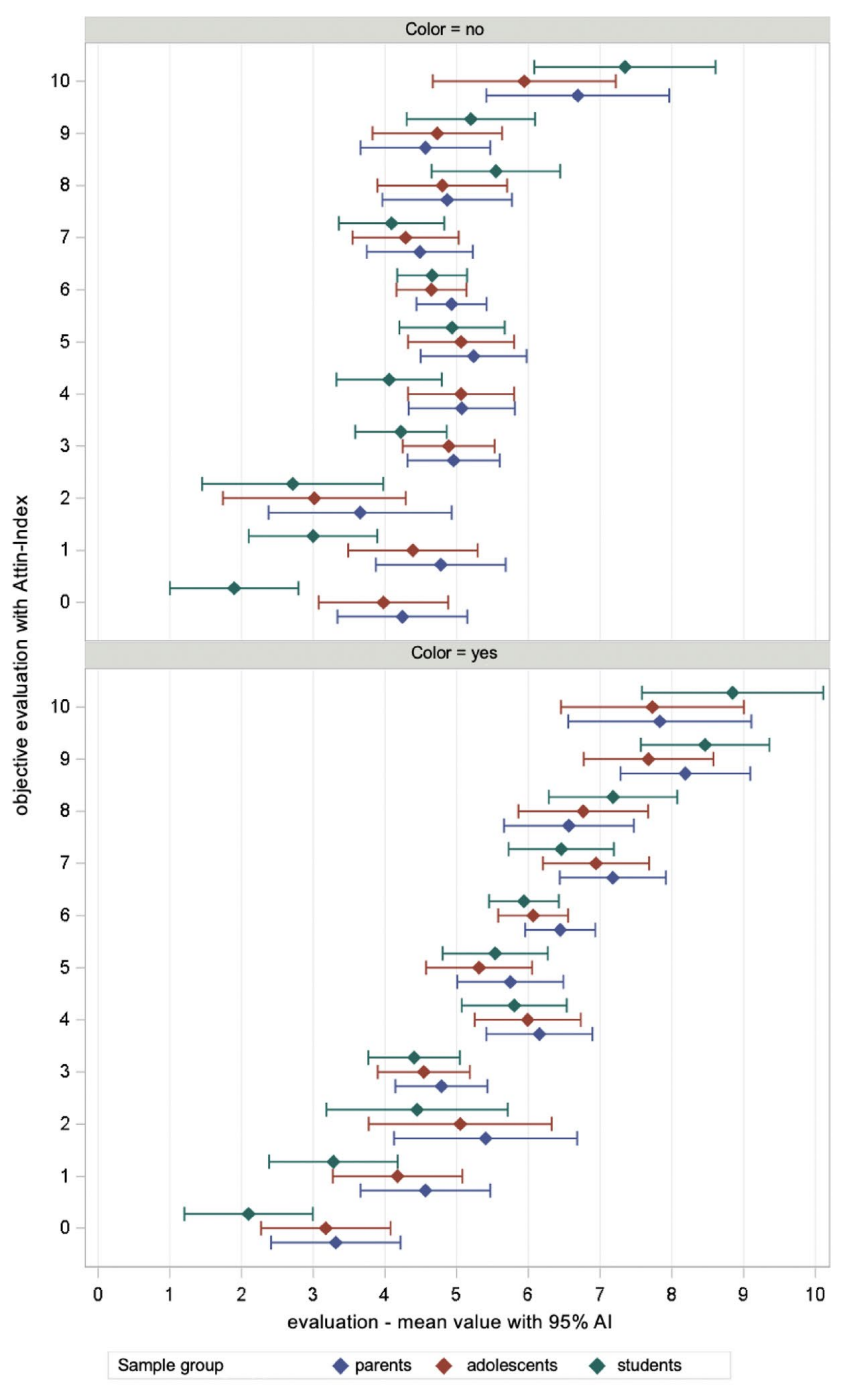
Influence of a plaque disclosing agent on the subjective evaluation of oral hygiene. Comparison between parents (blue), adolescents (red) and students (green). It was differentiated between records with the use of a plaque disclosing agent (lower) and native records (upper) and sorted according to the objective evaluation of oral hygiene with the Attin-index.

Figure 8 summarizes the evaluation of oral hygiene depending on the use of a plaque disclosing agent. The use of a plaque disclosing agent seemed to lead to a more precise assessment among parents, adolescents, and students.

Without the use of a plaque disclosing agent, oral hygiene was rated as significantly cleaner. On average, the subjective evaluation was 0.26 points (95% CI [− 0.57; 0.06]) lower than the objective evaluation without plaque disclosing agent and 0.96 point (95% CI [0.64; 1.27]) higher with plaque disclosing agent (*p* < 0.0001). The objective assessment, meaning the degree of oral hygiene presented, had a significant influence on the deviation from the objective assessment (*p* < 0.0001) with the mean deviation ranging from 3.25 point too high (95% CI [2.45; 4.06]) for Attin = 0 to 2.46 too low (95% CI [− 3.58; 1.34]) too low for Attin = 10. There is a significant interaction between the objective assessment and the use of a plaque disclosing agent (*p* < 0.0001).

### Time to first fixation in an area of interest

Areas of interest were defined and the time to first fixation in an area of interest was recorded. Not all participants reached fixation in an area of interest on all photos, therefore time-to-events methods were used for analysis. We found that compared with Attin = 4, all other evaluations of oral hygiene were associated with longer times to first fixation. The differences for Attin = 7 and Attin = 8 were significant, with HR 1.50 (95% CI [1.13, 2.00], *p* = 0.005) and HR 1.42 (95% CI [1.03, 1.95], *p* = 0.032) respectively. Staining has no significant association with the time to first fixation (HR 1.02, 95% CI [0.96, 1.08], *p* = 0.5). Adolescent patients (HR 1.77, 95% CI [1.14, 2.76], *p* = 0.011) and students (HR 1.61, 95% CI [1.02, 2.54], *p* = 0.043) had longer times to first fixation than the patients’ parents.

### Association between cleaning behavior and rating

A mixed linear model was used to test the association between the brushing behavior of the adolescents and their evaluation of the photographs, with the application of a plaque disclosing agent, brushing technique, brushing duration and objective evaluation as fixed factors, and photograph and subject as random factors. The median brushing time was 78.5 s. The maximum brushing time was 125 s. The shortest brushing time was 35 s.

**Fig. 9:**
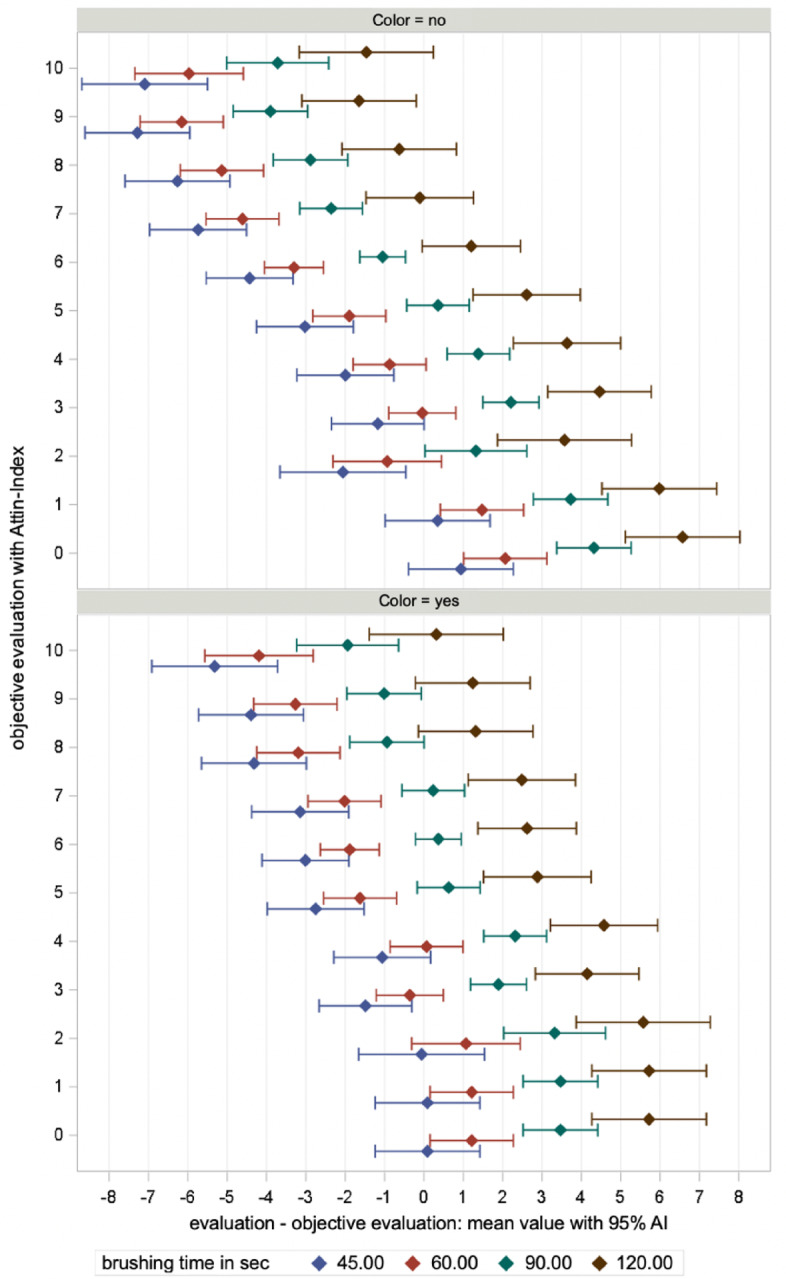
Deviation of the subjective evaluation of oral hygiene from the objective, sorted according to the comparison of brushing time of adolescents (blue = 45 s, red = 60 s, green = 90 s, brown = 120 s). It was differentiated between records with the use of a plaque disclosing agent (lower) and native records (upper) and sorted according to the objective evaluation of oral hygiene with the Attin-index.

Although it seemed that subjects with shorter brushing times tended to rate oral hygiene as better than subjects with longer brushing times, this effect was not significant (*p* = 0.16 for linear term and *p* = 0.10 for quadratic term). Brushing technique also did not show a significant association with the outcome (Fig. 9).

In addition, it was investigated how the adolescent test subjects rated their own photographs, and whether there was a deviation from the objective assessment of oral hygiene using the Attin index.

There appears to be a tendency for patients with very good to good oral hygiene, meaning their photographs received a rating of 0–3 during the objective evaluation, to rate their native photographs more strictly. Patients with an objective rating between 4 and 6 tended to rate their oral hygiene as better than that. All patients with an objective rating of 7 or higher rated their oral hygiene as cleaner than it is.

**Fig. 10:**
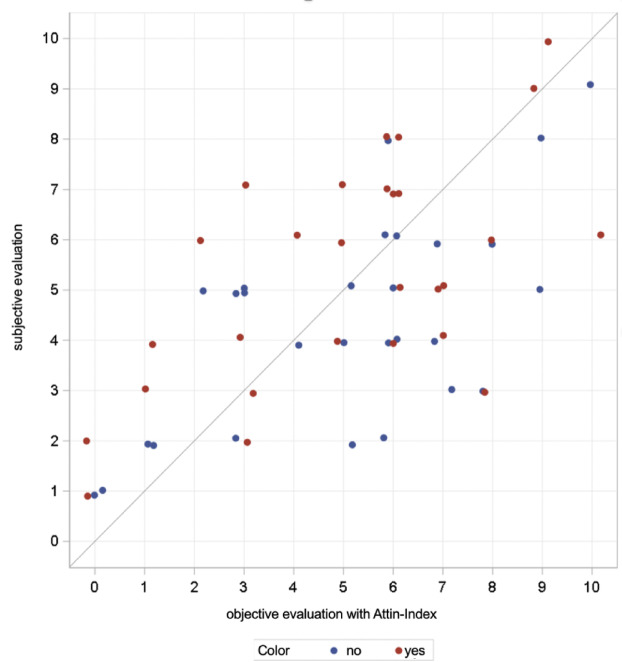
Deviation of the subjective assessment of the objective evaluation with the Attin-index. It is differentiated between records with the use of a plaque disclosing agent (red) and native records (blue).

In the subject groups with very good and very poor oral hygiene, meaning 0–3 and 7–10, it appears that the assessment of their oral hygiene does not change significantly with the use of a plaque disclosing agent. In the middle rating group of 4–6, the overall assessment was stricter while evaluating images with disclosed plaque (Fig. 10).

## Discussion

### Visual path length

On average, parents had a longer visual path on average, while students had the shortest visual path overall.

Students seemingly analyzed the photograph systematically according to a pattern: determining AOIs, identifying them as such and continuing to the next area. In the visual path analysis the lay persons, especially the parents, showed a rather chaotic visual pattern. They started at one point, jumped to the contralateral side to take in a different area, returned to the previous area; the visual path was continued in this elusive pattern. This could point to an insecurity concerning the evaluation of the observed, and thus a reference was searched for orientation.

Furthermore, the degree of presented oral hygiene had a significant influence on the visual path length. With a larger amount of plaque, more areas were examined. The use of a plaque disclosing agent apparently had a similar effect through which the visual paths of all groups increased in length. The improved visibility led to an increased plaque identification and a lengthening of the visual path length by side by side comparisons. A direct comparison of these insights concerning the visual path length is difficult. In studies showing similar numbers of images and a comparable viewing duration per record this data was not analyzed^24,25^.

### Evaluation of oral hygiene

The evaluation of participants was dependent on the degree of presented oral hygiene. The perception of good or bad oral hygiene differed between the groups due to experience. The use of a plaque disclosing agent presented a significant influence. The staining of the plaque led to a more strict or rather precise evaluation of oral hygiene.

A study by Mensi et al.^26^, investigating the use of plaque disclosing agents in professional plaque removal, showed that using a plaque disclosing agent, an additional 60% of plaque reduction was achieved compared to plaque removal without it. Plaque disclosing agents thus have an influence on recognizing plaque and support hygiene measures. In a children’s home in Japan, a study with adolescents revealed that the use of plaque disclosing agent led to an adaption of the brushing behavior and improved plaque reduction^27^. In further studies, these insights could be confirmed^28,29^.

Additionally, it was recognized that the group of parents had a tendency for a stricter and more accurate evaluation of oral hygiene, suggesting benefits in parental support and check ups on their children’s oral hygiene measures.

Apart from this, it is questionable whether parents can serve as role models due to their superior knowledge, or if they themselves show deficits in their oral hygiene performance. In the parallel-performed studies by Deinzer et al.^30^ and Eidenhardt et al.^31^, the brushing behavior of 66 parent–child-pairings was recorded. Parents and children seemed to stick to the brushing time, but the quality of hygiene measures concerning technique and the cleaning of all tooth surfaces differed. Parents were a role model, albeit a bad one, since children sometimes showed similar deficits.

Furthermore, a weak but not significant correlation was observed in this study between the brushing time, brushing behavior, and the evaluation. Adolescents exhibiting better brushing behavior, especially concerning brushing duration, tended to evaluate oral hygiene more accurately, including in their own photographs.

Other studies investigating the self-assessment of teeth^29,32,33^ focused on the contentment with one’s own teeth and their look as well as their influence on quality of life. It was also examined if there were any cross-relations between the self-assessment of oral hygiene and the discontentment with one’s own body^34^.

## Limitations

To conduct this eye-tracking study, intraoral photographs were used for the evaluation rather than direct clinical examinations, which would be difficult to perform with lay person investigators. Of course, static images under experimental conditions may differ from natural clinical observation.

The expert group consisted of dental students in their final semester of dental school rather than more experienced investigators, which might affect the generalizability of our findings.

## Conclusion

The eye-tracking-technology presents a reliable method for an objective comparison of oral hygiene record evaluation. This study was able to show that the visual perception of oral hygiene by a panel of adolescents, parents, and dental students differed from each other. The group of students evaluated the degree of presented oral hygiene the most accurately, which was to be expected of the expert group. The parents gave a stricter evaluation than their children, and their involvement in their children’s oral hygiene might help support the result of brushing.

The group of adolescents appeared more benevolent in their judgement. When comparing the adolescents among each other, a weak correlation between longer brushing time and more accurate oral hygiene evaluation was observed.

The use of a plaque disclosing agent presented a great support in the evaluation of oral hygiene and, when used at home, might help to optimize the brushing behavior.

## Data Availability

The datasets used and analyzed during the current study are available from the corresponding author on reasonable request.
